# p53, p21, and cyclin d1 protein expression patterns in patients with breast cancer

**DOI:** 10.14202/vetworld.2021.2833-2838

**Published:** 2021-10-31

**Authors:** Marwa Mohammed Ali Jassim, Khetam Habeeb Rasool, Majid Mohammed Mahmood

**Affiliations:** 1 Department of Basic Science, College of Dentistry, Al-Muthanna University, Al-Muthanna, Iraq; 2 Department of Biology, College of Science, Mustansiriyah University, Baghdad, Iraq.

**Keywords:** breast cancer, D1 protein, Iraq, P21, P53

## Abstract

**Background and Aim:**

The mutation in the wild-type tumor suppressor gene p53 is the most common genetic change in human tumors. In addition, the normal function of p21, which is both antiproliferative and an inhibitor of the cell cycle, is disrupted in some types of cancer. Meanwhile, cyclin D1 is a member of the cyclin protein family that is involved in regulating cell cycle progression. This study aimed to assess the expressions of the cell cycle inhibitory proteins p21, cyclin D1, and tumor suppressor gene p53, as well as their influence on the expressed histopathological changes in breast cancer tissues.

**Materials and Methods:**

Overall, 40 breast tissue specimens were investigated in this study, 30 of which were cancerous, while 10 were healthy tissues. p53, p21, and cyclin D1 expression patterns were detected using an immunohistochemistry (IHC) system.

**Results:**

The IHC reactions for p53 were positively observed in 27/30 (90%) cancerous tissues, compared with 2/10 (20%) normal breast tissues. For p21, reactions were observed in 28/30 (93.33%) cancerous tissues and 3/10 (30%) control tissues. For cyclin D1, reactions were observed in 25/30 (83.33%) cancerous tissues and 1/10 (10%) control tissues. The differences between the breast cancer tissues and the control tissues were statistically highly significant (p<0.01).

**Conclusion:**

The high expression rates of p21, cyclin D1, and p53 in malignant breast cancer cells with little or no regulatory role might imply mutational events in these proteins operating in concert with a variety of other genetic mutations in these tissues, which may play a molecular role in the development and/or progression of breast carcinogenesis.

## Introduction

Breast carcinogenesis is a multistage transformation emerging from a combination of hereditary and environmental factors that lead to the advanced accumulation of epigenetic and genetic alterations in the cells of breast tissue [1,2]. The annual incidence rate of breast cancer ranges from 1% to 2% in developed countries; in less developed countries, the increase in the incidence can be up to 5% from that of developed countries. Worldwide, it was expected that the number of new cases would increase from 10 to 15 million in 2020 [3]. The primary cancer suppressor gene is p53, which functions as a proliferation inhibitor and eliminator of anomalous cells, ultimately preventing tumor growth. Cellular stress is the main activator of p53, depending on the upstream regulatory kinase [4]. The mutation in the p53 gene remains the most common genetic change identified in human neoplasia. The functions of the gene can be lost, and cells in the G1/S phase will not arrest, resulting in the continuous replication of cells, which may result in the instability of genomes and the accumulation of deoxyribonucleic acid (DNA) mutations. Such events are associated with aggressive diseases and worse overall survival [5].

The cyclin-dependent kinase (CDK) inhibitor (p21) is the most important negative regulator of the cell cycle. In response to DNA damage, it is stimulated by p53 and arrests the cell cycle at the G1 phase to allow for DNA repair [6]. Furthermore, p21 can interact with several transcription factors, such as the inhibition of E2F protein complements and effects on cyclin/CDK complexes, to enhance the suppression of E2F genes and induce cell cycle arrest. Considered as a tumor suppressor, dysregulation of the p21 gene has been documented in various human tumors, including breast cancer [7]. The cell cycle is controlled by a family of cyclins and CDK through phosphorylation and dephosphorylation events. Cyclin D1 is produced during the G1 phase just before the checkpoint and plays the main role in the restriction (R) point regulation [8]. Elevated levels of G1 cyclins (D1, E), which are observed in some types of tumors, can result in uncontrolled cell proliferation. During the shift from G1 to S phase, cyclin D1 reaches the maximum level of expression and forms an active kinase complex with CDK4 or CDK6. The active cyclin/CDK complexes can then be regulated by binding to CDK inhibitors (p16 and p21), resulting in the inhibition of cell cycle progression from G1 to S phase [9,10].

This study aimed to investigate the expected roles of a number of cell cycle regulators, as well as a group of tumor suppressor genes, in Iraqi patients with breast carcinomas and compare their results to a healthy control group using modern molecular techniques. In addition, this study will evaluate the expression of these genes and their impact on the expressed histopathological alterations.

## Materials and Methods

### Ethical approval

The study was approved by Mustansiriyah University, College of Science, Department of Biology (Approval no.257).

### Study period and location

The study was conducted in March to August 2020. The block samples were collected from patients admitted to the surgical wards and from those who were subjected to biopsies and archived at the histopathological laboratories of the Medical City, Baghdad, Iraq, while the tests were conducted in the laboratories of the Medical College, University of Baghdad, Baghdad.

### Experimental design

#### Tissue processing and slide preparation

A total of 40 paraffin-embedded breast tissue blocks were investigated in this study, 30 of which belonged to females with breast cancer, while 10 belonged to females with normal breasts. The age of the women ranged between 40 and 60 years. Paraffin-embedded tissue samples from malignant and control tissues were subjected to serial sectioning at 4 μm thickness using a manual microtome with a specific microtome blade for each tissue block. One serial section was taken and mounted on an ordinary glass slide to confirm histopathological examination using hematoxylin and eosin staining, whereas other tissue sections mounted on positively charged slides were used for the following purposes.

The expressions of p53, cyclin D1, and p21 genes in the study groups were assessed using specific immunohistochemistry (IHC) using monoclonal rabbit anti-P53, anti-P21, and anti-cyclin D1 antibodies (cat. nos.: ab131442, ab109520, and ab16663), respectively, and targeting of nuclear specific proteins was performed following the IHC detection kit manufacturer’s instructions (Abcam, UK). The scoring and intensity of the signals were assessed using light microscopy (100×), according to the methods of Papamitsou *et al*. [11]. The primary concentrated antibodies were diluted to optimal concentration using phosphate-buffered saline (PBS, pH: 3.5); p21, cyclin D1, and p53 were diluted to 1:50.

#### Chromogen/substrate mixture

3,3’-Diaminobenzidine (DAB) chromogen (Abcam) (30 L) was added to DAB substrate (1.5 mL) and thoroughly mixed.

#### Preparation of slides

1. Each paraffin-embedded tissue block was sectioned to a thickness of 4 μm and mounted on charged slides
2. The sections were deparaffinized overnight in an oven at 60°C
3. The sections were rehydrated by serially dipping the slides in xylene (100%) twice for 15 min, ethanol (100%) twice for 5 min, ethanol (95%) once for 5 min, ethanol (70%) once for 5 min, ethanol (50%) once for 5 min, and PBS once for 5 min
4. The slides were allowed to dry at room temperature (25°C) for 5 min
5. In a dropwise manner, 0.3% H_2_O_2_ was added to cover the sections, which were incubated for 15 min before being rinsed twice in PBS buffer
6. The slides were immersed in a jar containing the epitope retrieval solution (sodium citrate buffer, pH 6), which was put in a water bath at 95°C for 20-25 min
7. The slides were washed twice with gentle agitation in PBS, and tissue paper was used to wipe the surrounding area of the sections
8. After blocking the slides with protein block, they were incubated at 25°C for 1 h
9. The slides were washed once in PBS
10. Each slide was treated with 30-50 μL of the diluted primary antibody and incubated as directed by the manufacturer
11. The slides were washed thrice in PBS
12. Complement was applied, and the slides were incubated at 25°C for 20 min
13. The slides were washed twice in PBS
14. HRP conjugate was added to the slides, which were incubated at 25°C for 40 min
15. The slides were rinsed 4 times in PBS
16. The slides were incubated for 10 min at 25°C with a combination of DAB chromogen and DAB substrate
17. The slides were rinsed 4 times with PBS
18. Counterstaining was applied to the tissue for 3-5 min before being rinsed for 2 min under running tap water
19. The slides were dehydrated and cleared by serial dipping in ethanol (50%) once for 5 min, ethanol (70%) once for 2 min, ethanol (95%) once for 2 min, ethanol (100%) twice for 2 min, ethanol (100%) twice for 2 min, ethanol (100%) twice for 2 min, and xylene (100%) twice for 2 min
20. A mounting solution was used to mount the slides.

### Statistical analysis

The statistical analysis was performed using SPSS program version 21.0 (IBM, NY, USA). Chi-square test and odds ratios were used to evaluate the significant differences between the study groups. The differences among the studied groups were considered statistically significant at p<0.05.

## Results

### p53 expression

**Figure-1:**
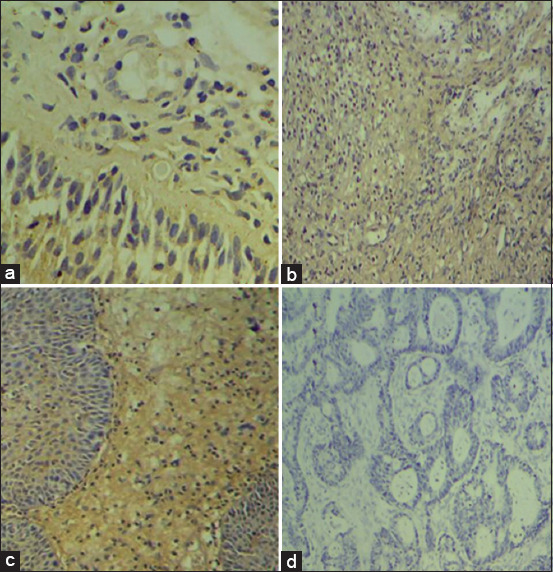
Representative images of breast carcinomas tissues staining for p53 (a), p21 (b), and cyclin D1 (c) expression patterns. These panels represent positive cases for each protein expression pattern that appeared as a brown discoloration at the nuclear and cytoplasmic locations, whereas panel (d) portrays a tumor in which the corresponding protein is not expressed.

Figure-1 displays a micrograph of p53 IHC-positive signals in the breast carcinoma tissues, which were observed as a brownish discoloration with nuclear and cytoplasmic localization under high-power field analysis.

The results of the p53 IHC signal intensity and its scoring distribution in the breast tissues are shown in Table-1. Reactions with low (1+), moderate (2+), and high (3+) signal scores of p53 IHC reactions were expressed in 5 (16.67%), 9 (30%), and 13 (43.33%) samples, respectively, while no signal was expressed in 3 (10%) samples.

**Table-1:** Distribution of P53-IHC signal scoring and intensity in healthy and cancerous breast tissues.

P53-IHC signal	Scoring			Stain	Intensity		
	
	Healthy control	Breast carcinoma	Pearson’s Chi-square (p-value)		Healthy control	Breast carcinoma	Pearson’s Chi-square (p-value)
Negative			p=0.00 highly sign. (p<0.01)	No stain			p=0.027 sign. (p<0.05)
n	8	3		n	8	3	
%	80.0	10.0		%	80.0	10.0	
+				Weak			
n	1	5		n	2	4	
%	10.0	16.67			20.0	13.33	
++				Moderate			
n	1	9		n	0	6	
%	10.0	30.0		%	0.0	20.0	
+++				Strong			
n	0	13		n	0	17	
%	0.0	43.33		%	0.0	56.67	
Total				Total			
n	10	30		n	10	30	
%	100.0	100.0		%	100.0	100.0	
Odds ratio	3.271			Odds ratio	3.271		

–=Highly significant difference at p<0.01 and significant at p<0.05 using Pearson’s Chi-square test. +=Referred to low p53 signal score, ++=Moderate signal score, +++=Strong signal score

In comparison, strong p53 IHC reactions were not observed in healthy tissues, with low (1+) and moderate (2+) signals collectively occurring in 20% of the samples, while no signal was observed in the rest (80%). The results of the odds ratio showed that the breast cancer samples had the highest value (3.271). Overall, there were significant variations (p<0.01) between the examined groups.

### p21 expression

The results of the signal scoring and strength of p21 expression in the breast cancer tissues are shown in Table-2 (for cancerous ones) as well as control tissues. In the breast cancer group, high (3+), low (1+), and moderate (2+) signals for p21 IHC reactions were expressed in 56.67%, 23.33%, and 13.33% of the samples, respectively, while there was no signal in 6.67% of the samples. The results of the odds ratio were revealed to be 1.604. In healthy tissues, low (1+) and moderate (2+) signals for p21 IHC reactions were observed in 20% and 10% of the samples, respectively, while no sample expressed a strong (3+) signal score. In addition, no signal was observed in the rest of the samples (70%).

**Table-2:** The P21-IHC distribution of signal scoring and intensity in healthy and cancerous breast tissues.

P21-IHC signal	Scoring			Stain	Intensity		
	
	Healthy control	Breast carcinoma	Pearson’s Chi-square (p-value)		Healthy control	Breast carcinoma	Pearson’s Chi-square (p-value)
Negative			p=0.007 highly sign. (p<0.01)	No stain			p=0.041 sign. (p<0.05)
n	7	2		n	7	2	
%	70.0	6.67		%	70.0	6.67	
+				Weak			
n	2	7		n	3	6	
%	20.0	23.33		%	30.0	20.0	
++				Moderate			
n	1	4		n	0	10	
%	10.0	13.33		%	0.0	33.33	
+++				Strong			
n	0	17		n	0	12	
%	0.0	56.67		%	0.0	40.0	
Total				Total			
n	10	30		n	10	30	
%	100.0	100.0		%	100.0	100.0	
Odds ratio	1.604			Odds ratio	1.604		

–=Highly significant difference at p<0.01 and significant at p<0.05 using Pearson Chi-square test. +=Referred to low p21 signal score, ++=Moderate signal score, +++=Strong signal score

### Cyclin D1 expression

The results of IHC staining of cyclin D1 are shown in Table-3. Moderate (2+), low (1+), and high signals for cyclin D1 IHC reactions were found in 33.33%, 13.33%, and 36.67% of the samples, respectively, while no signal was found in the rest of the samples (16.67%). In healthy tissues, a moderate (2+) signal was expressed in one sample (10%), while low (1+) and high (3+) signals for cyclin D1 reactions were not detected in any sample. In addition, 90% of these samples did not have any signal. The differences among the studied groups were statistically significant (p<0.05). The results of the odds ratio reached 2.351. Protein expression patterns are of particular importance in the development of breast cancer, and IHC has provided tremendous benefits in assessing the predictive and developmental markers in cancer.

**Table-3:** The immunohistochemical results of cyclin D1 signal scoring and intensity in healthy and cancerous breast tissues.

Cyclin D1–IHC signal	Scoring			Stain	Intensity		
	
	Healthy control	Breast carcinoma	Pearson’s Chi-square (P-value)		Healthy control	Breast carcinoma	Pearson’s Chi-square (p-value)
Negative			p=0.047 sign. (p<0.05)	No stain			p=0.039 sign. (p<0.05)
n	9	5		n	9	5	
%	90.0	16.67		%	90.0	16.67	
+				Weak			
n	0	4		n	1	6	
%	0.0	13.33		%	10.0	20.0	
++				Moderate			
n	1	10		n	0	9	
%	10.0	33.33		%	0.0	30.0	
+++				Strong			
n	0	11		n	0	10	
%	0.0	36.67		%	0.0	33.33	
Total				Total			
n	10	30		n	10	30	
%	100.0	100.0		%	100.0	100.0	
Odds ratio	2.351			Odds ratio	2.351		

–=Highly significant difference at p<0.01 and significant at p<0.05 using Pearson’s Chi-square test. +=Referred to low cyclin D1 signal score, ++=Moderate signal score, +++=Strong signal score

## Discussion

The present study investigated the abnormal p53, p21, and cyclin D1 protein expression patterns in carcinomatous breasts. The findings showed that p53 is upregulated and has different scores in breast cancer tissues compared with the normal breast tissues. Archer *et al*. [12] studied the expression of p53 in advanced breast cancers and discovered that, out of 92 patients, 53 (57.6%) had positive outcomes. Similarly, reports by the previous studies have suggested that p53 overexpression is linked to high grades of breast cancer [13-16]. Increased expression of p53, according to Jin *et al*. [17], reflects the accumulation of wild-type p53 as a compensatory mechanism of the cell’s DNA damage and repair system; however, since mutated p53 protein is not digested as easily as wild-type protein inside tumor cells, it accumulates instead. As a result, high p53 expression can be used as a surrogate marker for p53 mutation.

Regarding p21, the majority of cases (28/30, 93.33%) showed positive immunostaining when compared with normal breast tissues (3/10, 30%), which is consistent with previous research [18,19]. The exact mechanisms of p21’s effect on oncogenesis and development remain unclear, and research on breast cancer has shown contradictory findings. In comparison to non-cancerous tissues, CDKN1A/p21 protein levels were found to be significantly higher in breast cancer tissues. There is mounting evidence that the role of p21 is linked to its cellular localization. When p21 is found in the cytoplasm, it acts as an oncogene, encouraging cell proliferation and progression through the cell cycle, whereas nuclear p21 has been implicated in pro-differentiating and senescence-inducing effects [20]. Furthermore, it has been documented that cells in breast and ovarian cancers often coexpress p21 and cyclin D1 genes, resulting in growth arrest [21]. As a result, the coexpression of cell cycle inducers and inhibitors may suggest that these tumors retain key aspects of canonical cell cycle regulation.

Cyclin D1 positivity was also detected in the majority (25/30, 83.33%) of breast cancer sections compared with normal breast sections (1/10, 10%). It is an important regulator of the cell cycle that performs a central role in the pathogenesis of cancer and determines uncontrolled cellular proliferation; the cyclin D1 gene (*CCND1*) is amplified in approximately 20% of mammary carcinomas. Recently, *in vitro* and *in vivo* studies have established cyclin D1 as a controller of cellular invasiveness and aggressiveness. Cyclin D1 overexpression is a key determinant of the reciprocal interaction between cancer cells and the stroma, resulting in a “tumor-promoting” effect [22]. Assessing the role of each feature of cyclin D1 in cancer progression could aid in the development of therapies that are more precisely targeted and customized.

Breast carcinogenesis is considered to be accelerated by aberrant cyclin D1 overexpression, which is mediated by the cell cycle. It has been shown that inducing cyclin D1 is necessary to complete the cell cycle in cells arrested in the early G1 process by binding to a CDK4/CDK6 and inactivating the retinoblastoma protein in the cell cycle [21,22].

The current study used IHC to examine p53, p21, and cyclin D1 expressions in breast tissue in an Iraqi population, and the study confirmed their essential roles in the cellular events associated with breast carcinogenesis. However, the sample size should be increased in the future to better understand and validate our results, which is of promising importance in the axes of prediction, diagnosis, treatment, and follow-up of breast cancer.

## Conclusion

The high expression rates of p21, cyclin D1, and p53 in malignant breast cancer cells with little or no regulatory role might imply mutational events in these proteins operating in concert with a variety of other genetic mutations in these tissues, which may play a molecular role in the development and/or progression of breast carcinogenesis.

## Authors’ Contributions

MMAJ and MMM: Planned and conceptualized the study. MMAJ, MMM, and KHR: Carried out the experiments, analyzed the data, performed the laboratory analysis, as well as the curation of the data. MMAJ and MMM: Wrote the draft of the manuscript. KHA: Reviewed and edited the manuscript. All authors read and approved the final manuscript.
